# Potential for Recurrent Mpox Outbreaks Among Gay, Bisexual, and Other Men Who Have Sex with Men — United States, 2023

**DOI:** 10.15585/mmwr.mm7221a1

**Published:** 2023-05-26

**Authors:** Emily D. Pollock, Patrick A. Clay, Adrienne Keen, Dustin W. Currie, Rosalind J. Carter, Laura A. S. Quilter, Adi V. Gundlapalli, Jonathan Mermin, Ian H. Spicknall

**Affiliations:** ^1^National Center for HIV, Viral Hepatitis, STD, and TB Prevention, CDC; ^2^Center for Forecasting and Outbreak Analytics, CDC; ^3^Center for Global Health, CDC; ^4^National Center for Immunization and Respiratory Diseases, CDC; ^5^Office of Public Health Data, Surveillance, and Technology, CDC.

More than 30,000 monkeypox (mpox) cases have been diagnosed in the United States since May 2022, primarily among gay, bisexual, and other men who have sex with men (MSM) (*1*,*2*). In recent months, diagnoses have declined to one case per day on average. However, mpox vaccination coverage varies regionally, suggesting variable potential risk for mpox outbreak recurrence (*3*). CDC simulated dynamic network models representing sexual behavior among MSM to estimate the risk for and potential size of recurrent mpox outbreaks at the jurisdiction level for 2023 and to evaluate the benefits of vaccination for preparedness against mpox reintroduction. The risk for outbreak recurrence after mpox reintroduction is linearly (inversely) related to the proportion of MSM who have some form of protective immunity: the higher the population prevalence of immunity (from vaccination or natural infection), the lower the likelihood of recurrence in that jurisdiction across all immunity levels modeled. In contrast, the size of a potential recurrent outbreak might have thresholds: very small recurrences are predicted for jurisdictions with mpox immunity of 50%–100%; exponentially increasing sizes of recurrences are predicted for jurisdictions with 25%–50% immunity; and linearly increasing sizes of recurrences are predicted for jurisdictions with <25% immunity. Among the 50 jurisdictions examined, 15 are predicted to be at minimal risk for recurrence because of their high levels of population immunity. This analysis underscores the ongoing need for accessible and sustained mpox vaccination to decrease the risk for and potential size of future mpox recurrences.

CDC adapted models of mpox transmission to estimate the risk for and size of potential mpox recurrences at varying levels of population-level mpox immunity (*4**,**5*). Immunity varied from 0%–99% in increments of approximately 4%. Immunity levels included persons who had received 1 or 2 vaccine doses or had a history of infection, which conveyed 37%, 67%, and 100% reduction in susceptibility to infection, respectively (*6*). At each immunity level modeled, 29%, 67%, and 4% of those with some immunity were assumed to have 1-dose, 2-dose, or infection-acquired immunity, respectively, with immunity concentrated among MSM with higher levels of sexual activity (*3*,*4*). Sensitivity analyses considered 65% and 83% reductions in susceptibility associated with receipt of 1 or 2 doses, respectively (*6*).

To model mpox reintroduction, five MSM with infectious mpox and high levels of sexual activity were introduced to the sexual network. Depending on the level of immunity and chance, introduced cases either initiated an outbreak (of variable size) or failed to sustain transmission. An outbreak was defined as a simulation with sustained mpox incidence 3 months after reintroduction. For each immunity level, model simulations were conducted until 50 simulated recurrent outbreaks occurred. This analysis assumed no additional vaccination after April 28, 2023, no behavioral adaptation (such as decreasing partner acquisition rates) among MSM in response to new mpox cases, and no loss of immunity due to demographic turnover during the 2-year period modeled.

Risk was assessed at the jurisdiction level for the 50 nonstate, Ending the HIV Epidemic (EHE) Initiative jurisdictions based on these results and each jurisdiction’s case and vaccine administration data, through April 2023 (*4**,**5*). These jurisdictions, many of which contain urban centers with large MSM populations, account for more than one half of all new HIV diagnoses.* The numerator for jurisdiction immunity level was the sum of persons who had received 1 and 2 doses of JYNNEOS vaccine and those who had already been infected, accounting for potential incomplete reporting (*2*,*3*,*5*). The denominator was based on the population at increased risk for *Monkeypox virus* exposure, estimated as the number of MSM aged ≥16 years who were recommended to receive HIV preexposure prophylaxis and the number of MSM aged ≥13 years living with HIV in each jurisdiction, using publicly available data (*7*,*8*). The estimated population was then increased by 25% for each jurisdiction to account for additional persons eligible for vaccination (e.g., MSM with lower levels of sexual activity than their already-eligible partners). Statistical models were fit to the simulated outbreak results to summarize the relationship between immunity level and both risk for and size of outbreak recurrence; each jurisdiction’s risk for and size of outbreak recurrence was then then inferred based on jurisdiction-specific immunity using these fitted curves. This activity was reviewed by CDC and was conducted consistent with applicable federal law and CDC policy.^†^

Conditional on mpox reintroduction into a jurisdiction, the risk for a recurrent mpox outbreak is linearly related to immunity: each percentage point increase in population immunity reduces outbreak risk by 0.62 percentage points across all immunity levels modeled (Figure 1). For example, Suffolk County, Massachusetts, with an estimated at-risk population immunity of 64%, has a 21% risk for a recurrent outbreak, whereas Harris County, Texas, with 17% immunity has a 50% risk, conditional on reintroduction (Table). No critical threshold to avert a recurrent mpox outbreak was identified; higher vaccination coverage among MSM at risk provides continued decreased recurrence risk across all immunity levels modeled. Sensitivity analyses using higher efficacy estimates of 65% and 83%, respectively, decreased the recurrence risk by 6 percentage points when population immunity was >20% (Supplementary Figure, https://stacks.cdc.gov/view/cdc/128422).

**FIGURE 1:**
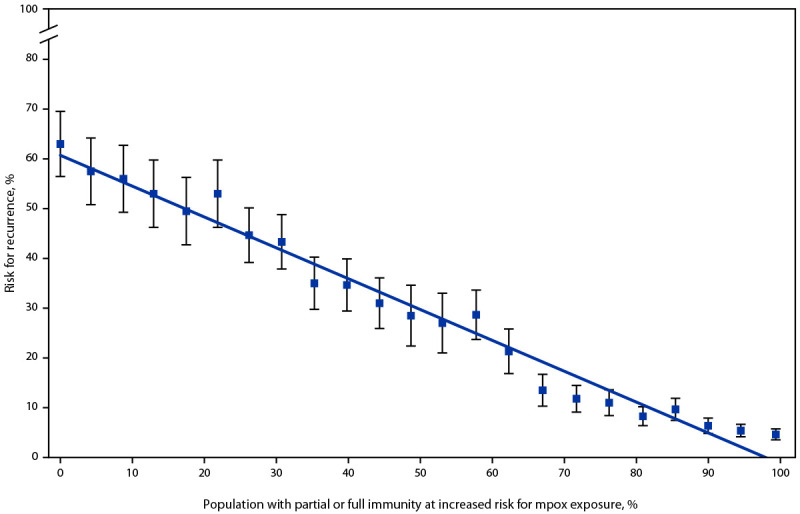
Risk* for recurrent mpox outbreak lasting >3 months, by immunity level^†^ — United States, 2023 **Abbreviations**: mpox = monkeypox; MSM = gay, bisexual, and other men who have sex with men. * Data points reflect simulated risk for a specific immunity level; line reflects predictions from linear model using immunity level as the independent variable and risk for recurrence as the dependent variable; error bars indicate 95% CIs assuming a binomial distribution based on the number of simulations required to produce 50 outbreaks. ^†^ Immunity was varied from 0% to 99% in increments of approximately 4%; at each level of immunity, 29%, 67%, and 4% of MSM with some immunity are assumed to have 1-dose, 2-dose, or infection-acquired immunity, conveying 37%, 67%, and 100% protection, respectively.

**TABLE:** Jurisdiction-specific estimates of immunity and inferred* risk and size of mpox recurrence — United States, 2023

Jurisdiction	Estimated immunity level, %^†^	Inferred* risk for recurrence, %	Inferred* cumulative *Monkeypox virus* infections vs. 2022^§^	Jurisdictional immunity grouping^¶^	MSM at increased risk for *Monkeypox virus* exposure**
Duval County, Florida	6	57	4.08	Low	12,425
Shelby County, Tennessee	10	55	3.77	Low	10,626
Hamilton County, Ohio	10	55	3.79	Low	9,970
Bexar County, Texas	11	54	3.67	Low	17,916
Dallas County, Texas	12	53	3.62	Low	45,264
Tarrant County, Texas	15	51	3.32	Low	15,909
Palm Beach County, Florida	15	52	3.36	Low	12,824
Hillsborough County, Florida	15	52	3.39	Low	17,802
Wayne County, Michigan	16	51	3.29	Low	14,705
Harris County, Texas	17	50	3.16	Low	60,769
San Bernardino County, California	18	49	3.07	Low	15,829
East Baton Rouge Parish, Louisiana	18	50	3.14	Low	3,735
Baltimore City, Maryland	19	49	3.04	Low	10,800
Pinellas County, Florida	20	48	2.96	Low	13,430
Gwinnett County, Georgia	21	48	2.89	Low	5,672
Marion County, Indiana	24	46	2.60	Low	12,681
Fulton County, Georgia	25	45	2.58	Low	27,831
Prince George's County, Maryland	26	44	2.03	Medium	9,007
Orange County, Florida	26	45	2.09	Medium	21,838
Dekalb County, Georgia	26	45	2.12	Medium	14,053
Cuyahoga County, Ohio	27	44	1.90	Medium	11,470
Cobb County, Georgia	27	44	1.96	Medium	5,980
Essex County, New Jersey	29	43	1.66	Medium	7,806
Franklin County, Ohio	31	42	1.41	Medium	15,752
Travis County, Texas	32	41	1.30	Medium	16,218
San Juan Municipio, Puerto Rico	32	41	1.30	Medium	3,773
Maricopa County, Arizona	32	41	1.33	Medium	33,513
Mecklenburg County, North Carolina	33	40	1.18	Medium	12,947
Montgomery County, Maryland	34	40	1.10	Medium	7,515
Clark County, Nevada	36	39	0.97	Medium	20,231
Bronx County, New York	36	39	0.98	Medium	19,723
Hudson County, New Jersey	37	38	0.86	Medium	8,009
Miami-Dade County, Florida	40	36	0.72	Medium	40,489
Orange County, California	45	33	0.48	Medium	17,090
Philadelphia County, Pennsylvania	47	32	0.42	Medium	18,771
Sacramento County, California	52	28	0.20	High	9,723
San Diego County, California	54	27	0.19	High	27,536
Riverside County, California	58	25	0.18	High	21,314
Broward County, Florida	59	24	0.18	High	33,886
Orleans Parish, Louisiana	61	23	0.18	High	8,057
Cook County, Illinois	63	22	0.17	High	60,444
Los Angeles County, California	63	22	0.17	High	117,361
Suffolk County, Massachusetts	64	21	0.17	High	10,356
King County, Washington	65	20	0.16	High	24,308
Alameda County, California	75	14	0.14	High	14,167
Queens County, New York	78	12	0.13	High	20,057
District of Columbia	98	<1	0.08	High	22,348
Kings County, New York	99	<1	0.07	High	30,540
New York County, New York	100	<1	0.07	High	37,900
San Francisco County, California	100	<1	0.07	High	23,577

**Abbreviations:** mpox = monkeypox; MSM = gay, bisexual, and other men who have sex with men.

* Risk and size of potential recurrence inferred from each jurisdiction's estimated immunity level based on primary simulated results.

^†^ At each level of immunity, 29%, 67%, and 4% of MSM with some immunity are assumed to have 1-dose, 2-dose, or infection-acquired immunity, conveying 37%, 67%, and 100% protection, respectively.

^§^ Median cumulative infections from simulations, relative to the size of the CDC-modeled 2022 mpox outbreak in the absence of additional vaccination or behavioral adaptation.

^¶^ Grouping based on thresholds identified in primary analysis. Threshold values are as follows: high >50%, medium = 25%–50%, and low <25%.

** Estimated as the number of MSM aged ≥16 years who were recommended to receive HIV preexposure prophylaxis and the number of MSM aged ≥13 years living with HIV in each jurisdiction, using publicly available data. The estimated population was then increased by 25% for each jurisdiction to account for additional persons eligible for vaccination (e.g., MSM with lower levels of sexual activity than their already-eligible partners).

In contrast to the linear relationship between increasing levels of immunity in the population at risk and the risk for a recurrent outbreak, immunity does have a threshold effect on the size of a recurrent outbreak (Figure 2). Three distinct jurisdictional groupings were identified: those with high, medium, and low immunity, defined as 50%–100%, 25%–49%, and <25% vaccine- or infection-induced immunity, respectively. High-immunity jurisdictions were predicted to experience small recurrences that resolved within 1 year. Medium-immunity jurisdictions were transitional, with more uncertainty: recurrence size increased exponentially and lasted 12–17 months. Finally, low-immunity jurisdictions were predicted to experience recurrences lasting 18–20 months that increased linearly in size with decreasing levels of immunity.

**FIGURE 2:**
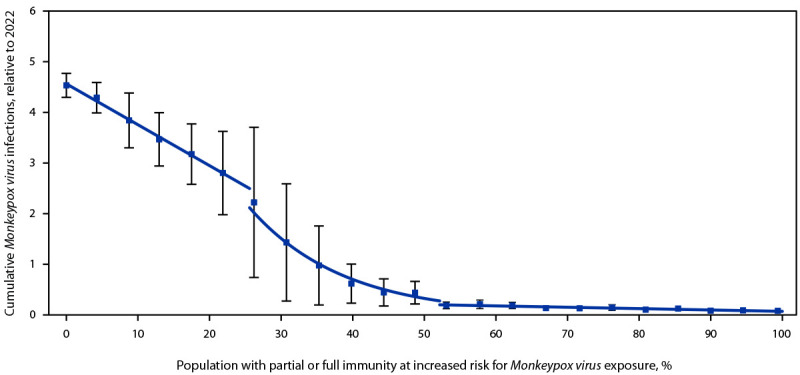
Cumulative *Monkeypox virus* infections* relative to 2022, by immunity level^†^ — United States, 2023 **Abbreviations**: mpox = monkeypox; MSM = gay, bisexual, and other men who have sex with men. * Median cumulative infections from simulations, measured among simulations in which an outbreak occurred, relative to the size of the CDC-modeled 2022 mpox outbreak; data points reflect simulated cumulative infection magnitude for a specific immunity level; lines reflect three separate linear model fits using immunity level as the independent variable and median cumulative incidence as the dependent variable among each threshold; first and last threshold were fit using estimates directly, and middle threshold was fit using log-transformed incidence estimates to reflect the rapid change in outbreak magnitude during this period; error bars indicate 25th–75th quartile observed across outbreaks. ^†^ Immunity was varied from 0% to 99% in increments of approximately 4%; at each level of immunity, 29%, 67%, and 4% of MSM with some immunity are assumed to have 1-dose, 2-dose, or infection-acquired immunity, conveying 37%, 67%, and 100% protection, respectively.

Overall, 44% of MSM at increased risk for *Monkeypox virus* exposure who live in EHE jurisdictions live in high-immunity jurisdictions that are likely to have minimal risk for recurrence, including Los Angeles, New York City, San Francisco, and Washington, DC (Table). However, 56% of MSM at increased risk for exposure who live in EHE jurisdictions live in low- or medium-immunity jurisdictions that are potentially at risk for mpox recurrences capable of sustained transmission should reintroduction occur (Table).

## Discussion

This analysis, which highlights the association between population mpox immunity and the risk for outbreak recurrence, underscores the need for accessible and sustained mpox vaccination services, particularly in communities with low vaccination coverage and among MSM at highest risk. Jurisdictions that achieved high vaccination coverage among populations at risk are not expected to experience large recurrences in the immediate future. Success in achieving high coverage in these jurisdictions was partially driven by early prioritization for vaccine distribution because of high early case counts when there was high vaccination demand. In other jurisdictions, vaccines became available after the outbreak had peaked and when demand had declined because of reduced perception of risk (*5*).

The findings in this report are subject to at least four limitations. First, to consider the downstream effects of mpox reintroduction and guide preparedness, mpox reintroduction was assumed to be certain. However, actual reintroduction risk will be influenced by global and local mpox infection dynamics. Although the intensity of the 2022 multinational outbreak has diminished substantially, areas with ongoing transmission remain. Attendance at large social engagements that attract domestic and international MSM travelers could result in reintroduction of mpox into local sexual networks. Assuming reintroduction is independent of subsequent outbreak recurrence risk, reintroduction has a proportional effect on recurrence: for example, if there were a 50% probability of reintroduction, the risk for recurrence would be 50% lower than that presented in the current analysis. In the absence of additional vaccination or mpox cases, immunity will decline in future years because of demographic turnover, thereby increasing recurrence risk. In addition, small outbreaks might occur even when the estimated risk for a large outbreak is relatively low. As an example, based on this model, Cook County, Illinois, has an estimated 22% risk for a sustained mpox recurrence; however, the city of Chicago, which is part of Cook County, has reported a new cluster of mpox cases that emerged in April 2023 (*9*). Second, inferred estimates of vaccine efficacy from Israel and the United States show a range of effectiveness (*6*). Conservative estimates for 1- and 2-dose efficacy of 37% and 67%, respectively, were used in the primary analysis, although sensitivity analysis examining higher efficacy did not appreciably change the results. Third, many of the sexual behavior data were derived from surveys conducted in 2012, and it is possible that patterns have changed over time and in response to recent disruptive public health events, including this mpox outbreak (*10*). The actual risk for and size of recurrent outbreaks might differ among the actual social and sexual engagements of MSM, which might also vary between communities. Finally, this analysis assumed no interventions took place in response to mpox reintroduction. However, this assumption highlights the benefits of vaccination for preparedness against mpox reintroduction. Delays between detection of reintroduction, mobilization of additional vaccination sites, vaccine administration, and immunologic protection are difficult to shorten, potentially leaving vulnerable communities at risk.

This analysis highlights the importance of public health programs identifying opportunities to promote vaccination before Pride-related and other events when vaccination interest might be higher, rather than vaccinating after reintroduction is identified. Focusing these vaccination efforts in low-coverage areas, and even in high-coverage areas, among MSM who are younger and newly sexually active and among groups with disproportionally low vaccination coverage, can help protect both individual persons and the entire community against a resurgence of mpox. CDC continues to recommend a full 2-dose course of the JYNNEOS vaccine for MSM and others at risk for *Monkeypox virus* exposure.

**Summary**

**What is already known about this topic?**

Monkeypox (mpox) has disproportionately affected gay, bisexual, and other men who have sex with men (MSM); the percentage of MSM with immunity due to vaccination or infection varies among jurisdictions.

**What is added by this report?**

Mathematical modeling suggests that the risk for future outbreaks depends linearly on the level of immunity in the population at risk; cumulative incidence, on the other hand, has multiple thresholds. More than 592,000 MSM live in jurisdictions with risk for mpox recurrences capable of sustained transmission if a cluster of infectious cases were reintroduced.

**What are the implications for public health practice?**

Increasing vaccination coverage among MSM at risk and in jurisdictions with low immunity has the potential to reduce the risk for and potential size of future mpox outbreaks.

## Footnotes

*
https://www.cdc.gov/endhiv/about.html

† 45 C.F.R. part 46, 21 C.F.R. part 56; 42 U.S.C. Sect. 241(d); 5 U.S.C. Sect. 552a; 44 U.S.C. Sect. 3501 et seq.
